# Giving a Hand: Synthetic Peptides Boost the Antifungal Activity of Itraconazole against *Cryptococcus neoformans*

**DOI:** 10.3390/antibiotics12020256

**Published:** 2023-01-27

**Authors:** Tawanny K. B. Aguiar, Ricardo M. Feitosa, Nilton A. S. Neto, Ellen A. Malveira, Francisco I. R. Gomes, Ana C. M. Costa, Cleverson D. T. Freitas, Felipe P. Mesquita, Pedro F. N. Souza

**Affiliations:** 1 Department of Biochemistry and Molecular Biology, Federal University of Ceará, Fortaleza 60451-970, Brazil; 2 Drug Research and Development Center, Department of Physiology and Pharmacology, Federal University of Ceará, Fortaleza 60430-275, Brazil

**Keywords:** azole drugs, combined activity, membrane pore formation, synthetic antifungal peptides

## Abstract

*Cryptococcus neoformans* is a multidrug-resistant pathogen responsible for infections in immunocompromised patients. Here, itraconazole (ITR), a commercial antifungal drug with low effectiveness against *C. neoformans*, was combined with different synthetic antimicrobial peptides (SAMPs), Mo-CBP3-PepII, RcAlb-PepII, RcAlb-PepIII, PepGAT, and PepKAA. The Mo-CBP3-PepII was designed based on the sequence of MoCBP3, purified from *Moringa oleifera* seeds. RcAlb-PepII and RcAlb-PepIII were designed using Rc-2S-Alb, purified from *Ricinus communis* seed cakes. The putative sequence of a chitinase from *Arabidopsis thaliana* was used to design PepGAT and PepKAA. All SAMPs have a positive liquid charge and a hydrophobic potential ranging from 41–65%. The mechanisms of action responsible for the combined effect were evaluated for the best combinations using fluorescence microscopy (FM). The synthetic peptides enhanced the activity of ITR by 10-fold against *C. neoformans*. Our results demonstrated that the combinations could induce pore formation in the membrane and the overaccumulation of ROS on *C. neoformans* cells. Our findings indicate that our peptides successfully potentialize the activity of ITR against *C. neoformans*. Therefore, synthetic peptides are potential molecules to assist antifungal agents in treating Cryptococcal infections.

## 1. Introduction

Fungal diseases are a threat to human health. From mild mycosis to severe lung infections, fungi affect over 300 million people worldwide, causing 1.6 million deaths annually [1]. In addition to the low number of new drugs available, in the past 40 years, an increasing resistance of these pathogens to traditional antifungal medications and treatments has been observed [2,3]. Thus, there is a need for new treatments for fungal diseases, which could be developed from new molecules exhibiting antifungal activity.

Among the deadliest fungal pathogens is the human-pathogenic yeast *C. neoformans*, a highly virulent yeast that can cause pneumonia and meningitis. *C. neoformans* is the leading cause of mortality among immunocompromised individuals, such as organ transplant patients and cancer patients undergoing chemotherapy [4]. *C. neoformans* virulence is a combination of some unique traits such as a polysaccharide capsule, which protects against phagocytosis; thermotolerance to 37 °C; the presence of melanin, protecting against UV light; and a variety of extracellular enzymes that act as defense mechanisms [1]. All these features, plus drug misuse over the years, has led to a rapid increase in drug resistance among the various strains [2], turning *C. neoformans* into a severe threat for human health.

The solution may lie in discovering drugs with mechanisms of action different from those of conventional drugs. Recently, synthetic antimicrobial peptides have emerged as potential candidates because they are based on natural antimicrobial peptides, but exhibit high activity and low toxicity levels [5,6,7]. In addition to being employed alone, synthetic peptides could be applied synergistically with commercial drugs to improve their action [8,9,10,11]. For example, Souza et al. [11] revealed that synthetic peptides improved the activity of griseofulvin against dermatophytes. By studying the mechanism of action, the authors revealed that synthetic peptides induced pore formation in the fungus membrane and increased the intracellular concentration of griseofulvin, which has a cytoplasmatic target [11]. 

Recently, our research group reported five synthetic peptides (Mo-CBP3-PepII, RcAlb-PepII, RcAlb-PepIII, PepGAT, and PepKAA), with higher activity against *C. neoformans* and which can induce pore formation in its membrane [7]. Those SAMPs have already been characterized, and the antifungal, antibacterial, and antiviral activity tested. The *Mo*-CBP3-PepII was designed based on the sequence of antifungal protein, MoCBP3, purified from *Moringa oleifera* seeds. *Rc*Alb-PepII and *Rc*Alb-PepIII were obtained from the sequence of a 2S albumin, Rc-2S-Alb, purified from *Ricinus communis* seed cakes. Lastly, PepGAT and PepKAA were designed from a putative sequence of a chitinase from *Arabidopsis thaliana*. All SAMPs have a positive liquid charge, hydrophobic potential ranging from 41–65%, secondary structure in an α-helix, and are cell-penetrating peptides [5,10,12]. This study aimed to evaluate the ability of those peptides to improve the activity of a commercial drug ITR against *C. neoformans.* Additionally, the mechanisms of action were assayed to understand the way in which this combined effect occurred. 

## 2. Materials and Methods

### 2.1. Biological Material and Chemicals

*C. neoformans* (ATCC 32045) was obtained by the Department of Biochemistry and Molecular Biology of the Federal University of Ceará (UFC), Fortaleza, Brazil. All the chemicals used in the experiments were obtained from Sigma Aldrich (São Paulo, SP, Brazil).

### 2.2. Synthetic Peptides

The synthetic peptides *Mo*-CBP3-PepII, *Rc*Alb-PepII, *Rc*Alb-PepIII, PepGAT, and PepKAA, were chemically synthesized by ChemPeptide (Shanghai, China) and were analyzed for purity and quality (≥95%) by reverse-phase high-performance liquid chromatography and mass spectrometry. 

### 2.3. Evaluation of Combined Antifungal Activity between SAMPs and ITR

The combined antifungal activity was evaluated as described in [11]. The combinations with SAMPs and itraconazole were constituted of peptides MIC_50_ [7] + itraconazole (ITR) 500 (1×) µg mL^−1^, peptides [5×D] + ITR [1×D], peptides [10×D] + ITR [5×D], and peptides [10×D] + ITR [10×D]. The cells were grown on YPD agar for approximately 15 days, and cryptococcal cells were then resuspended in YPD medium and standardized at 10^6^ cells mL^−1^. The assay was performed in 96-well plates and incubated for 24 h at 30 °C. Then, the absorbance was measured at 600 nm using an automated microplate reader (Epoch, Biotek, Santa Clara, CA, USA). The best combinations were used to study the mechanisms of action. All the controls were prepared using a solution of 5% DMSO in 0.15 M of NaCl (vehicle of SAMPs) and the solution of SAMPs and ITR alone.

### 2.4. Cell Membrane Integrity Assay

The cell membrane integrity of *C. neoformans* was evaluated as described in [7]. After incubation for 24 h, as described above, the samples were washed three times with sterile 0.15 M NaCl and centrifuged (5000× *g* 5 min at 4 °C) to remove the YPD medium. Then, the cells were incubated with propidium iodide (PI) for 30 min in the dark at room temperature (22 °C ± 2). Next, they were washed and centrifuged twice, under the same conditions as previously mentioned. Finally, the cryptococcal cells were observed with a fluorescence microscope (Olympus System Bx 60, Tokyo, Japan) using a 535 nm excitation and a 617 nm wavelength.

### 2.5. Overproduction of Reactive Oxygen Species (ROS)

The ROS overproduction was evaluated according to the method Dias et al. [12]. After incubation for 24 h, as described above, the samples were washed three times with sterile 0.15 M NaCl and centrifuged (5000× *g* 5 min at 4 °C) to remove the YPD medium. The analysis was conducted, as described, by PI assay. Next, 9 µL of 2′,7′ dichlorohfluorescein diacetate—DCFH-DA (Sigma, St. Louis, MI, USA)—was added and incubated in the dark for 30 min at room temperature (22 °C ± 2). Next, they were washed and centrifuged twice under the same conditions as those mentioned above. Then, the cryptococcal cells were observed under a fluorescence microscope (Olympus System BX 41, Tokyo, Japan), with an excitation wavelength of 488 nm and an emission wavelength of 525 nm.

### 2.6. 3/7 Caspase Activity

The caspase activity was measured after cell incubation for 24 h, in the presence and absence of synthetic peptides and ITR, according to the methodology described by Qorri and Harless [13]. After incubation for 24 h, as described above, the samples were washed three times with sterile 0.15 M NaCl and centrifuged (5000× *g* 5 min at 4 °C) to remove the YPD medium. The samples were washed and centrifuged as described above, and the cells were incubated with 3 μL of CellEvent^®^ (ThermoFisher, São Paulo, SP, Brasil) for 30 min in the dark at room temperature (22 °C ± 2). Then, the samples were washed and centrifuged again. Finally, the cryptococcal cells were observed under a fluorescence microscope (Olympus System BX60), with an excitation wavelength of 342 nm and emission wavelength of 441 nm. 

### 2.7. Counting Fluorescent Cells Using ImageJ

The fluorescent *C. neoformans* cells were counted using ImageJ software using the Cell Counter plugin, according to the methodology described by Aguiar et al. [7]. The cells were counted in the bright and fluorescent field of the same picture. Cells presenting fluorescence were called positive cells, and those without fluorescence were called negative cells. The number of cells in the bright field was considered 100%. The cells were calculated using the equation: number of positive cells × 100/number of cells in the bright field. The number of negative cells was calculated following the equation: number of cells in the bright field—number of positive cells. Three different images were used to count the cells. 

### 2.8. Statistical Analysis

Experiments were performed in three biological replications, and each biological replication was performed with three technical replicates. The statistical analyses were performed using GraphPad Prism (version 5.01) for Microsoft Windows. All data obtained in the assays were compared using standard deviation and the one-way analysis of variance (ANOVA), followed by the Tukey test (*p* < 0.05).

## 3. Results

### 3.1. Combined Anticryptococcal Activity of Synthetic Peptides Combined with ITR

The hypothesis that peptides could be employed to improve the anticryptococcal activity of ITR was supported by previous results published by our research group that peptides induce pore formation on *C. neoformans* membranes and thus could increase the intracellular concentration of ITR. Aguiar et al. [7] showed that PI could move through the membrane of *C. neoformans* cells treated with peptides. This result suggests the pore formation in the membrane but could also be mistaken for an increase in membrane permeability, rather than actual pore formation. Because of this, the authors provided a new experiment using FITC-Dextran with a size of 6-kDa. The movement of FITC-Dextran through the membrane of *C. neoformans* treated cells really indicates the presence of pores induced by peptides, which was corroborated by scanning electron microscopy [7]. Based on this, it was believed that peptides could improve the action of ITR. 

The method used to evaluate the activity between synthetic peptides and ITR was that developed by Souza et al. [11]. All the concentrations without dilution [1×] used to produce the combination were defined by Aguiar et al. [7], and 5-fold dilution [5×D] and 10-fold dilution [10×D] combinations were created following Souza et al. [11]. In total, were performed 36 combinations between all the synthetic peptides and ITR, with three dilutions (Table 1). Here, we choose to use the MIC50 concentration as defined by Aguiar et al. [7], for two reasons: (1) we already have all the concentration of peptides obtained by a previous screening, and these concentrations are known to be effective; (2) if we employ the MIC100, it could be difficult to see the real combined activity and the mechanism of action.

For all experiments, a solution made of 5% DMSO in 0.15 M NaCl (DMSO-NaCl) was used as a control (Table 1). At the concentration of 500 µg mL^−1^ [1×], ITR inhibited only 45.3% of *C. neoformans* growth (Table 1). This concentration of ITR to reach the MIC50 was previously defined against this isolated of *C. neoformans* by Aguiar et al. [7]. At concentrations of 100 [5×D] and 50 [10×D] µg mL^−1^, the inhibitory activity of ITR against *C. neoformans* dropped to 12.2 and 4.5%, respectively (Table 1). All synthetic peptides presented the MIC_50_ at 25, 0.04, 0.04, 0.04, and 0.04, respectively, for Mo-CBP3-PepII, RcAlb-PepII, RcAlb-PepIII, PepGAT, and PepKAA (Table 1). As expected, the inhibitory activity of all synthetic peptides against *C. neoformans* was affected at concentrations of 5×D and 10×D dilutions (Table 1).

Regarding the combinations made by synthetic peptides and ITR, the threshold was established to choose the best combinations. Only the combinations with an inhibitory activity ≥80% were considered (Table 1). Based on that, the best combinations were Mo-CBP3-PepII [5×D] + ITR [5×D], Mo-CBP3-PepII [10×D] + ITR [5×D], RcAlb-PepII [1×] + ITR [1×], RcAlb-PepII [5×D] + ITR [5×D], and RcAlb-PepIII [5×D] + ITR [5×D], as they inhibited, respectively, 84.1, 87.2, 83.9, 82.3, and 84% of the growth of *C. neoformans* (Table 1). All combinations presented great results; however, the best combination was Mo-CBP3-PepII [10×D] + ITR [5×D], which inhibits the growth of *C. neoformans* by 87.2% (Table 1). Alone, Mo-CBP3-PepII [10×D] and ITR [5×D] presented an inhibition, respectively, of 2.1 and 12.2% of *C. neoformans* growth (Table 1). All the best combinations were further used to understand the mechanism of action of combined activity. 

### 3.2. Membrane Pore Formation on C. neoformans Cells

**Figure 1:**
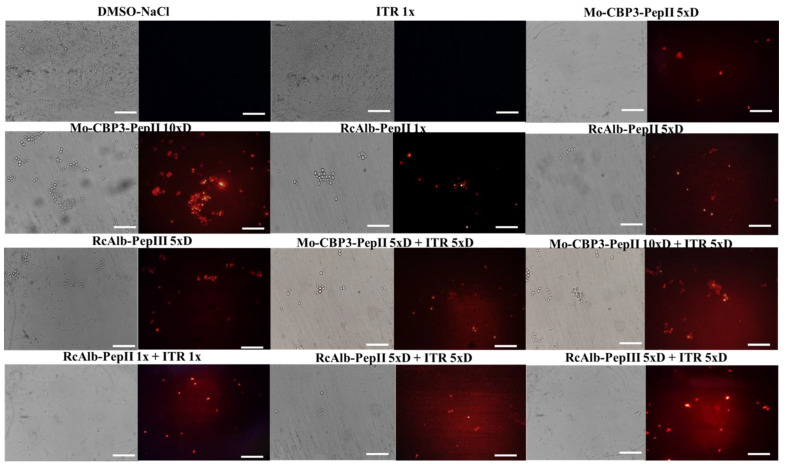
Fluorescence images showing membrane pore formation on *C. neoformans* cells. Membrane pore formation was measured using a propidium iodide uptake assay. Bars: 100 μm; ITR: itraconazole. 1× is the solution without dilution; 5×D is the solution 5-times diluted; 10×D is the solution 10-times diluted.

The first mechanism analyzed was the ability to induce pore formation on *C. neoformans* cells, evaluated by the propidium iodide (PI) uptake assay. The PI uptake is based on a release of red fluorescence, which results from the interaction between PI and a cell’s DNA. However, PI can only pass through a damaged membrane, and control membranes do not allow PI’s movement. Therefore, red fluorescence indicates damage to the membrane. As expected, *C. neoformans* cells treated with DMSO-NaCl control solution presented no red fluorescence, indicating no pores on the membranes (Figure 1). The same result was found in cells treated with ITR 1× (Figure 1). Even with diluted concentrations and a reduction in activity (Table 1), all synthetic peptides Mo-CBP3-PepII 5×D, Mo-CBP3-PepII 10×D, RcAlb-PepII 1×, RcAlb-PepII 5×D, RcAlb-PepIII 5×, alone, induced pore formation on *C. neoformans* cells (Figure 1).

As expected, all combinations of peptides and ITR induced pore formation in the membrane of *C. neoformans* cells (Figure 1). However, interestingly, all combinations presented a higher number of cells with red fluorescence, except for Mo-CBP3-PepII 10×D (Figure 1). It is important to notice that brightfield optical microscopy showed a small number of cells in combinations when compared to the controls. This is also a significant result and a confirmation of the data presented in Table 1 (Figure 1).

### 3.3. ROS Overaccumulation and Apoptosis in C. neoformans Cells

**Figure 2:**
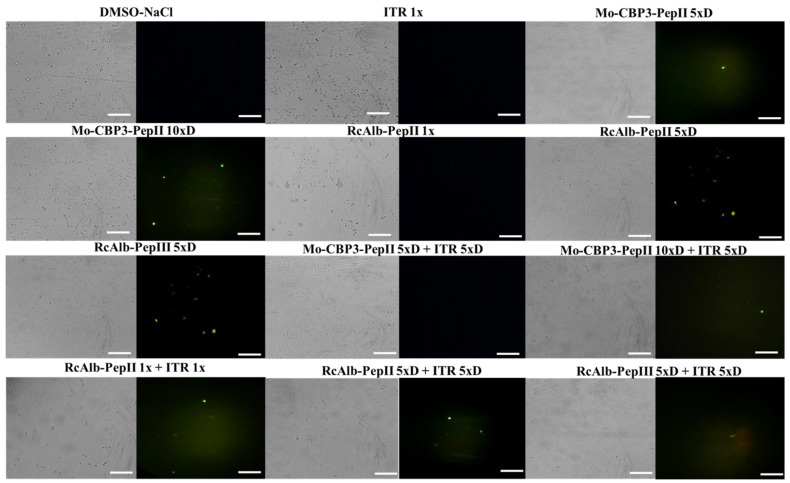
Fluorescence images showing the formation of ROS overaccumulation on *C. neoformans* cells. ROS overaccumulation was measured by DCFH-DA assay. Bars: 100 μm; ITR: itraconazole. 1× is the solution without dilution; 5×D is the solution 5-times diluted; 10×D is the solution 10-times diluted.

**Figure 3:**
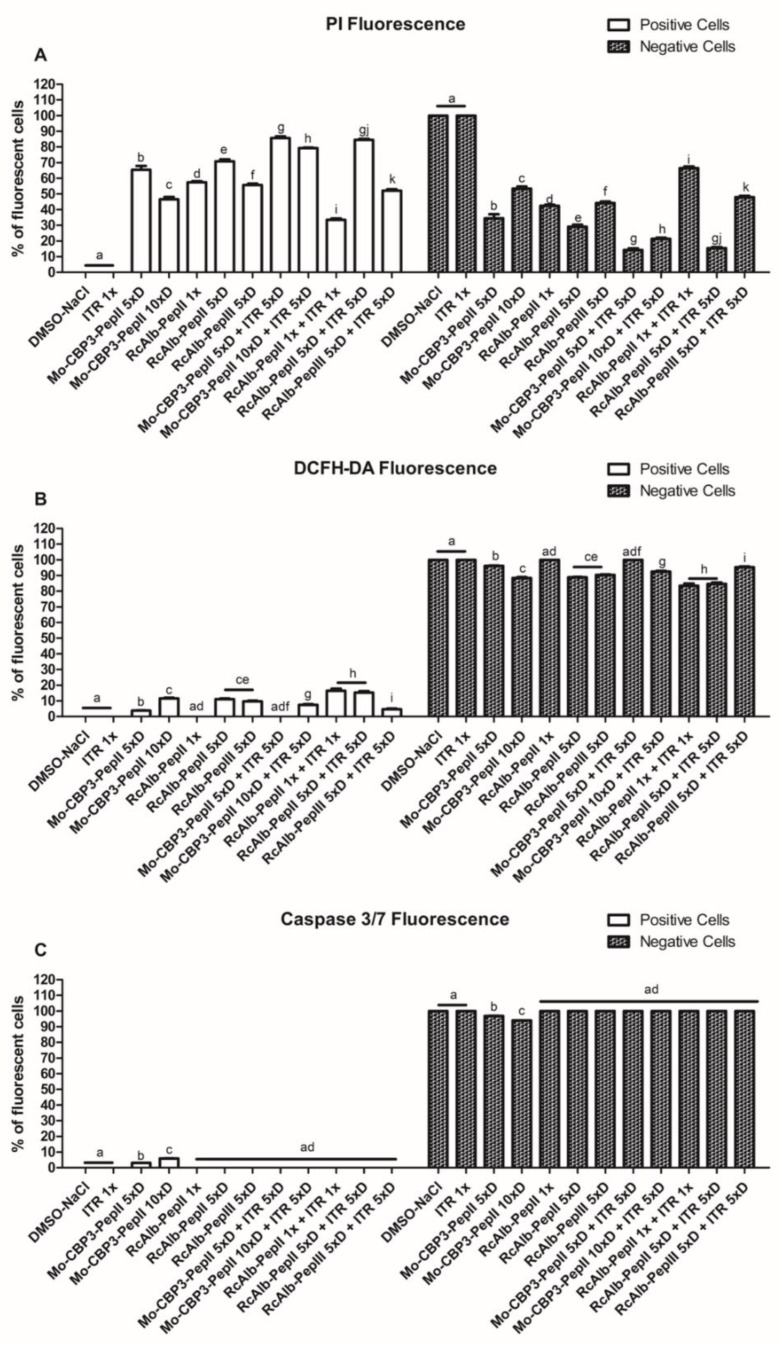
The number of fluorescent *C. neoformans* cells PI (**A**), DCFH-DA (**B**), and caspase 3/7 (**C**). The letters represent the mean ± standard deviation of three replicates. Different lowercase letters indicate statically significant difference compared to DMSO-NaCl by analysis of variance (*p* < 0.05).

In addition to pore formation, the ability to induce ROS overproduction and apoptosis was also evaluated (Figure 2 and Figure 3). The control cells treated with DMSO (Figure 2) and ITR (Figure 2) presented no ROS accumulation. Among the peptides alone, only RcAlb-PepII 5×D (Figure 2) did not induce ROS overaccumulation in *C. neoformans* cells. Regarding the combination, only the combination of Mo-CBP3-PepII 5×D + ITR 5×D (Figure 2) did not induce over-accumulation. Interestingly, the combination of RcAlb-PepIII 5×D + ITR 5×D (Figure 2) induced ROS overaccumulation. The analysis of the brightfield optical microscopy showed a small number of cells in combinations when compared to the controls. This is also a significant result and a confirmation of the data presented in Table 1 (Figure 2).

Regarding apoptosis induction, only *C. neoformans* cells treated with Mo-CBP3-PepII 5×D and Mo-CBP3-PepII 10×D (Supplementary Figure S1) presented green fluorescence, as indicative of apoptosis, even though the fluorescence was slight. None of the other treatments induced apoptosis in *C. neoformans* cells. In this case, the best information came from light field microscopy, which showed a small number of cells in the combinations when compared to the controls. This is also a significant result and a confirmation of the data presented in Table 1 (Figure 3). The counting of cells corroborated the qualitative analysis. 

### 3.4. Counting C. neoformans Fluorescent Cells

By analyzing images using ImageJ software, it was possible to evaluate the number of PI-fluorescent cells in each treatment (Figure 3A). The combinations made by *Mo*-CBP_3_-PepII 5×D + ITR 5×D, *Mo*-CBP_3_-PepII 10×D + ITR 5×D, and *Rc*Alb-PepII 5×D + ITR 5×D presented a higher number of positive PI-fluorescent cells than their respective controls (Figure 3A). *Mo*-CBP_3_-PepII 5×D + ITR 5×D, *Mo*-CBP_3_-PepII 10×D + ITR 5×D, and *Rc*Alb-PepII 5×D + ITR 5×D presented a total of 85, 74, and 84%, respectively, positive fluorescent cells. In contrast, ITR, *Mo*-CBP_3_-PepII 5×D, *Mo*-CBP_3_-PepII 10×D, *Rc*Alb-PepII 5×D alone presented a total of, respectively, 0, 45, 64, 70% PI-fluorescent cells (Figure 3A).

Regarding the DCFH-DA-fluorescent cells, the behavior was different. In this case, the combinations made by RcAlb-PepII 1× + ITR 1× and RcAlb-PepII 5×D + ITR 5×D (Figure 3B) presented a higher number of DCFH-DA-fluorescent cells than did the controls. RcAlb-PepII 1× + ITR 1× and RcAlb-PepII 5×D + ITR 5×D presented, respectively, a total of 16 and 15% DCFH-DA-fluorescent cells. On the other hand, the control ITR, RcAlb-PepII 1×, and RcAlb-PepII 5×D presented, respectively, a total of 0, 0, and 8% DCFH-DA-fluorescent cells. In this case, for the solution of RcAlb-PepII 1× + ITR 1×, the ROS accumulation is a result of the combined action of the peptide and ITR (Figure 3B).

## 4. Discussion

Cryptococcal disease caused by *C. neoformans* has a high impact on public health worldwide. For example, cryptococcal meningitis causes 181,000 deaths yearly, with a dramatic mortality rate of 100% without the correct treatment [14]. In addition to this alarming problem, the arsenal of drugs to treat infection caused by *C. neoformans* is quite limited. Cryptococcosis infections are treated based on antifungal drugs such as polyenes, pyrimidine analogs, and azoles. To date, one new class of antifungal drugs, echinocandins, has been developed, but its effects against *C. neoformans* are disappointing [14]. 

Azoles such as ITR are gain attention again in the treatment of cryptococcal infection. Azoles such as ITR exhibit a mechanism of action based on the inhibition of ergosterol biosynthesis. The inhibition of the ergosterol synthesis seems to be caused by the formation of a complex between ITR and the heme group iron of the fungal cytochrome P450, which leads to the inhibition of lanosterol 14α-demethylase [14,15]. Several studies have shown the potential of azoles, such as ITR and fluconazole (FLU), to treat infection caused by *C. neoformans* [16,17,18,19,20,21]. Currently, cryptococcal meningitis therapy uses azole drugs such as FLU. However, in cases in which FLU cannot be given because of intolerance or toxicity, ITR is an acceptable alternative [21,22]. Another advantage of ITR is that it is promising for treating cryptococcosis in patients with and without acquired immunodeficiency syndrome [19].

The development of resistance to ITR by *C. neoformans* produced the need to seek alternatives to continue employing ITR in treatment. One of these alternatives is the combination of ITR with other drugs to improve its activity. The combination of drugs can prevent the emergence of pathogen-resistance [8,9,11,16,17,23]. Some recent studies tried to combine other classes of drugs, such as anti-inflammatories [17], aminoglycosides [16], and even synthetic peptides [8,9,11], with antifungal drugs to enhance their action against *C. neoformans*. 

For example, Shrestha et al. [16] reported the combined activity against *C. neoformans* of the aminoglycoside K20 with ITR. Alone, K20 and ITR presented activity against *C. neoformans* at 4 and 780 µg mL^−1^. In contrast, when combined, the concentration to reach the same activity decreases to 187 and 1 for K20 and ITR [16]. In another study, Rocha et al. [17] evaluated the synergistic effect between ibuprofen and ITR against *C. neoformans*. The authors discussed that alone, the MIC50 of ibuprofen against *C. neoformans* was 512 µg mL^−1^ and for ITR, it was 500 µg mL^−1^ [17]. However, the combination between them reduced the concentrations of ibuprofen and ITR, respectively, to 16 and 125 µg mL^−1^ [17]. The use of synthetic peptides in synergism with an azole is still poorly studied, even against *C. neoformans*. As far as we know, the most recent studies regarding synthetic peptides improving the activity of azoles and polyenes came from our research group [8,9,11].

Recently, two synthetic peptides bioinspired in the *Mo*-CBP_3_ protein of *Moringa oleifera* have a synergistic antifungal effect with itraconazole. The peptides *Mo*-CBP_3_-PepI and *Mo*-CBP_3_-PepIII were able to act in synergy with ITR on *Candida ssp.* biofilms, with a mechanism of action involving pore formation and overproduction of reactive oxygen species [8]. Another study revealed that synthetic peptides PepGAT and PepKAA, designed from a chitinase from *Arabidopsis thaliana*, enhanced the activity of ITR against the biofilm of *C. albicans* by 10 times [9]. 

Here, we tested different combinations of synthetic peptides and ITR against *C. neoformans*. Our results show that synthetic peptides enhanced the antifungal activity of ITR against *C. neoformans* at low concentrations (Table 1). The synthetic peptides Mo-CBP_3_-PepII, RcAlb-PepII, RcAlb-PepIII, PepGAT, and PepKAA used in this study have already presented high activity and showed a studied mechanism of action against *C. neoformans* [7]. Our results revealed that synthetic peptides induced membrane pore formation, DNA degradation, and apoptosis in *C. neoformans* cells. Among all combinations and peptides, the peptides Mo-CBP_3_-PepII, RcAlb-PepII, and RcAlb-PepIII were able to improve the activity of ITR, even at a concentration 5 times lower than MIC50 (Table 1). 

Aguiar et al. [7] reported that the MIC50 of Mo-CBP_3_-PepII against *C. neoformans* was 25 µg mL^−1^. The results presented in this study revealed that even at a concentration of 2.5 µg mL^−1^ [10×D], Mo-CBP_3_-PepII was able to improve the activity of ITR at a concentration of 100 µg mL^−1^ [5×D] (Table 1). It is noteworthy to mention that, alone at this concentration, neither exhibit any activity (Table 1). Indeed, this was the best combination found in this study. Another good result was found for RcAlb-PepII and RcAlb-PepIII. Both have an MIC50 against *C. neoformans* at 0.04 µg mL^−1^ [7]. Here, at a concentration of 0.008 µg mL^−1^ [5×D], both were able to enhance the activity of ITR at 5×D of its MIC50 concentration (Table 1). 

As discussed above, the mechanism of action of ITR is based on the inhibition of ergosterol biosynthesis by inhibiting the activity of lanosterol 14α-demethylase [14]. One of the mechanisms of resistance to ITR in *Cryptococcus* spp. is associated with mutations in ERG11, the gene responsible for encoding lanosterol 14α-demethylase. However, other resistance mechanisms include efflux pumps that decrease the intracellular level of drugs and reduce membrane permeability to ITR [14]. 

Aguiar et al. [7] revealed that at the MIC50concentration, all peptides induced pore formation in the *C. neoformans* membrane, as revealed by the PI uptake assay. Surprisingly, at diluted concentrations and alone, all peptides induced membrane pore formation (Figure 2), as revealed by the PI uptake assay. The pores formed by peptides in the membrane of *C. neoformans* might lead to a higher concentration of ITR inside the cells, improving its activity. This may explain how peptides enhanced the activity of ITR, even at a low concentration, such as the 5×D concentration (Table 1). Indeed, cells of *C. neoformans* treated with a combined solution made of peptides and ITR, present a higher number of cells with red fluorescence (Figure 2).

Another important point is that the combinations made of peptides and ITR can also maintain higher levels of ROS (Figure 2) than can the treatment with peptides alone. High levels of ROS are lethal to cells because ROS interact with protein, DNA, and lipids, causing loss of function [24]. Additionally, the induction of apoptosis mediated by 3/7 caspase was investigated (Figure 3). However, our results revealed that it is not a mechanism employed by the combination of peptides and ITR. 

The reduction in the concentration of ITR with higher activity is a significant result of this study. However, another important point needs to be discussed. It has already been posted that the same synthetic peptides presented here can reduce the toxicity of ITR to human red blood cells (HRBC) [8,9]. As is known, all drugs present collateral effects on the patient, and ITR is not an exception. Some side effects such as headache, dizziness, vomiting, diarrhea, cardiotoxicity, and hypertension effects [25] have been noted. In our previous study, ITR at a concentration of 1000 µg mL^−1^ alone caused 100% hemolysis of A-, B-, and O-type HRBC [8,9]. However, when combined with the same synthetic peptides tested here, the hemolysis rate decreased to levels below 10% [8,9]. Here, the peptides raised the activity of ITR with a reduced concentration of 100 µg mL^−1^.

## 5. Conclusions

Here, synthetic peptides were able to increase the activity of ITR against *C. neoformans* with a concentration 5 times lower than the MIC50. This combined effect occurs because peptides increase the cytoplasmic concentration of ITR by improving its movement through the *C. neoformans* membrane. Therefore, synthetic peptides are potential molecules for clinical application as adjuvants to commercial drugs that are rapidly becoming useless.

## Figures and Tables

**Table 1:** Combined antifungal activity between synthetic peptides and ITR against *C. neoformans*.

Treatments	% of Inhibition of *C. neoformans* Growth
DMSO-NaCl	0
ITR 1×	45.3 ± 0.021
ITR 5×D	12.2 ± 0.003
ITR 10×D	0
*Mo*-CBP_3_-PepII 1× (MIC_50_)	50.0 ± 0.004
*Mo*-CBP_3_-PepII 5×D	12.4 ± 0.001
*Mo*-CBP_3_-PepII 10×D	0
*Rc*Alb-PepII 1× (MIC_50_)	50.0 ± 0.001
*Rc*Alb-PepII 5×D	2.6 ± 0.005
*Rc*Alb-PepII 10×D	0
*Rc*Alb-PepIII 1× (MIC_50_)	50.0 ± 0.004
*Rc*Alb-PepIII 5×D	10.6 ± 0.003
*Rc*Alb-PepIII 10×D	0
PepGAT 1× (MIC_50_)	50.0 ± 0.009
PepGAT 5×D	17.1 ± 0.003
PepGAT 10×D	0
PepGAT 1× (MIC_50_)	50.0 ± 0.005
PepGAT 5×D	20.4 ± 0.009
PepGAT 10×D	0
*Mo*-CBP_3_-PepII 1× + ITR 1×	78.8 ± 0.004
*Mo*-CBP_3_-PepII 5×D + ITR 1×	74.5 ± 0.008
*Mo*-CBP_3_-PepII 10×D + ITR 1×	73.8 ± 0.009
*Mo*-CBP_3_-PepII 5×D + ITR 5×D	84.1 ± 0.001
*Mo*-CBP_3_-PepII 10×D + ITR 5×D	87.2 ± 0.002
*Mo*-CBP_3_-PepII 5×D + ITR 10×D	71.7 ± 0.005
*Mo*-CBP_3_-PepII 10×D + ITR 10×D	70.8 ± 0.006
*Mo*-CBP_3_-PepII 1× + ITR 1×	78.8 ± 0.002
*Rc*Alb-PepII 1× + ITR 1×	83.9 ± 0.001
*Rc*Alb-PepII 5×D + ITR 1×	74.8 ± 0.006
*Rc*Alb-PepII 10×D + ITR 1×	65.6 ± 0.007
*Rc*Alb-PepII 5×D + ITR 5×D	82.3 ± 0.002
*Rc*Alb-PepII10×D + ITR 5×D	63.5 ± 0.005
*Rc*Alb-PepII 5×D + ITR 10×D	71.7 ± 0.004
*Rc*Alb-PepII 10×D + ITR 10×D	70.8 ± 0.003
*RcAlb-*PepIII 1× + ITR 1×	73.8 ± 0.004
*RcAlb-*PepIII 5×D + ITR 1×	74.5 ± 0.001
*RcAlb-*PepIII 10×D + ITR 1×	69.8 ± 0.01
*RcAlb-*PepIII 5×D + ITR 5×D	84.1 ± 0.02
*RcAlb-*PepIII 10×D + ITR 5×D	73.9 ± 0.009
*RcAlb-*PepIII 5×D + ITR 10×D	66.3 ± 0.001
*RcAlb-*PepIII 10×D + ITR 10×D	49.0 ± 0.008
PepGAT 1× + ITR 1×	73.4 ± 0.02
PepGAT 5×D + ITR 1×	69.4 ± 0.005
PepGAT 10×D + ITR 1×	66.3 ± 0.011
PepGAT 5×D + ITR 5×D	59.0 ± 0.02
PepGAT 10×D + ITR 5×D	54.2 ± 0.001
PepGAT 5×D + ITR 10×D	52.8 ± 0.008
PepGAT 10×D + ITR 10×D	47.4 ± 0.005
PepKAA 1× + ITR 1×	68.6 ± 0.003
PepKAA 5×D + ITR 1×	71.4 ± 0.004
PepKAA 10×D + ITR 1×	69.6 ± 0.05
PepKAA 5×D + ITR 5×D	45.6 ± 0.002
PepKAA 10×D + ITR 5×D	50.3 ± 0.015
PepKAA 5×D + ITR 10×D	50.3 ± 0.025
PepKAA 10×D + ITR 10×D	50.6 ± 0.009

ITR 1×, 5×D, and 10×D refer, respectively, to 500, 100, and 50 µg mL^−1^. 500 a 100 50. Mo-CBP3-PepII 1×, 5×D, and 10×D refer, respectively, to 25, 5, and 2.5 µg mL^−1^. RcAlb-PepII 1×, 5×D, and 10×D refer, respectively, to 0.04, 0.008, and 0.004 µg mL^−1^. RcAlb-PepIII 1×, 5×D, and 10×D refer, respectively, to 0.04, 0.008, and 0.004 µg mL^−1^. PepGAT 1×, 5×D, and 10×D refer, respectively, to 0.04, 0.008, and 0.004 µg mL^−1^. PepKAA 1×, 5×D, and 10×D refer, respectively, to 0.04, 0.008, and 0.004 µg mL^−1^. The highlighted combinations were the best found and used in the mechanisms of action.

## Data Availability

The data supporting this study’s findings are available on request from the corresponding author.

## Supplementary Materials

The following supporting information can be downloaded at: https://www.mdpi.com/article/10.3390/antibiotics12020256/s1, Figure S1: Fluorescence images showing 3/7-mediated apoptosis n on *C. neoformans* cells.
