# Supplementary material for: Giving a Hand: Synthetic Peptides Boost the Antifungal Activity of Itraconazole against Cryptococcus neoformans
Source: Antibiotics (Basel). 2023 Jan 27;12(2):256. doi: 10.3390/antibiotics12020256 (PMC9952215; doi:10.3390/antibiotics12020256)
Supplement: Supplementary file 1 [file antibiotics-12-00256-s001.zip › antibiotics-2147730-supplementary.pdf]

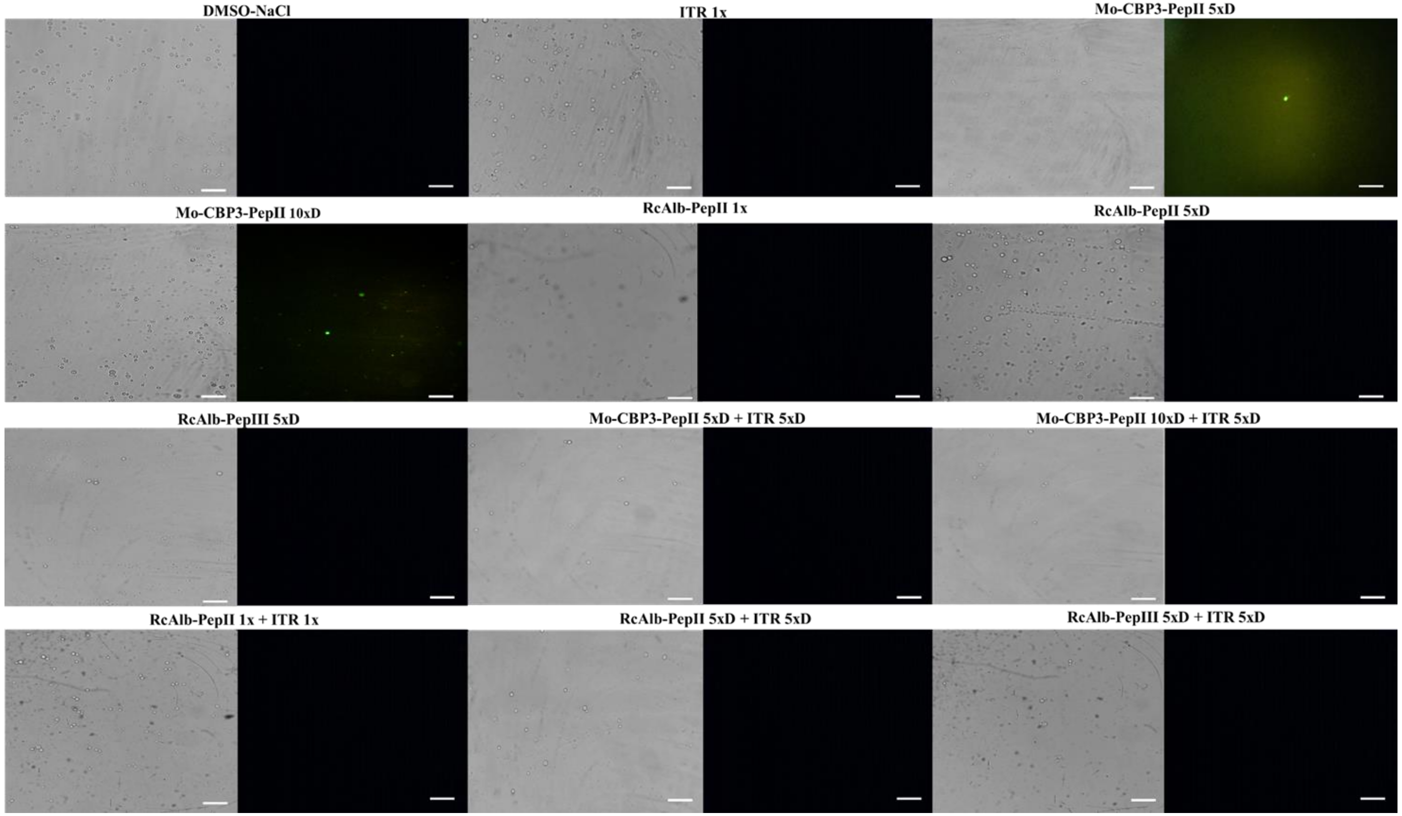

Supplementary Figure S1. Fluorescence images showing 3/7-mediated apoptosis n on *C. neoformans* cells. Apoptosis was measured by CellEvent ki assay. Bars: 100 µm; ITR: Itraconazole. 1x is the solution without dilution; 5xD is the solution 5-times diluted; 10xD is the solution 10-times diluted.
